# Associations of Sedentary Behaviour, Physical Activity, Cardiorespiratory Fitness and Body Composition with Risk of Sleep-Related Breathing Disorders in Children with Overweight/Obesity: A Cross-Sectional Study

**DOI:** 10.3390/jcm9051544

**Published:** 2020-05-20

**Authors:** Lucia V. Torres-Lopez, Cristina Cadenas-Sanchez, Jairo H. Migueles, Mireia Adelantado-Renau, Abel Plaza-Florido, Patricio Solis-Urra, Pablo Molina-Garcia, Francisco B. Ortega

**Affiliations:** 1 PROFITH “PROmoting FITness and Health through Physical Activity” Research Group, Sport and Health University Research Institute (iMUDS), Department of Physical Education and Sports, Faculty of Sport Sciences, University of Granada, 18011 Granada, Spain; cristina.cadenas@uca.es (C.C.-S.); jairohm@ugr.es (J.H.M.); abeladrian@ugr.es (A.P.-F.); patricio.solis.u@gmail.com (P.S.-U.); pablomolinag5@gmail.com (P.M.-G.); ortegaf@ugr.es (F.B.O.); 2 MOVE-IT research group, Department of Physical Education, Faculty of Education Sciences, University of Cádiz, 11519 Cádiz, Spain; 3 Biomedical Research and Innovation Institute of Cádiz (INiBICA) Research Unit, Puerta del Mar University Hospital, University of Cádiz, 11009 Cádiz, Spain; 4 LIFE Research Group, University Jaume I, 12071 Castellon, Spain; adelantm@uji.es; 5 IRyS Research Group, School of Physical Education, Pontificia Universidad Católica de Valparaíso, Valparaíso 2374631, Chile; 6 Department of Rehabilitation Sciences, KU Leuven, University of Leuven, 3000 Leuven, Belgium; 7 Department of Biosciences and Nutrition, Karolinska Institutet, 14183 Huddinge, Sweden

**Keywords:** sleep quality, preadolescents, childhood obesity, sedentarism, aerobic capacity, obstructive sleep apnea

## Abstract

The aim of this study was to examine the associations of sedentary behaviour, physical activity, cardiorespiratory fitness (CRF), and body composition parameters with risk of sleep-related breathing disorders (SRBD) in children with overweight/obesity. One-hundred and nine children (10.0 ± 1.1 years old, 45 girls) with overweight (*n* = 27) and obesity (*n* = 82) were included. Television viewing time was self-reported by using the Spanish adaptation of the “Youth Activity Profile” (YAP) questionnaire. Sedentary time and physical activity were measured with accelerometry. CRF was assessed with the 20-m shuttle-run test and body composition parameters with Dual-energy X-ray absorptiometry. SRBD were evaluated by using the Spanish version of the Pediatric Sleep Questionnaire. Television viewing time was positively associated with risk of SRBD (*r* = 0.222, *p* = 0.021). CRF was negatively correlated with risk of SRBD (*r* = −0.210, *p* = 0.030). Body composition parameters were positively associated with risk of SRBD (all *p* < 0.05), except fat mass index. Stepwise regression analyses showed that body mass index (BMI) explained the largest proportion of the variance in SRBD (*r*^2^ = 0.063, *p* = 0.01) and television viewing time was the only one added after BMI (*r*^2^ change = 0.048, *p* = 0.022). This study supports the notion that higher body weight status negatively influences risk of SRBD and adds that unhealthy behaviours could contribute to worsen SRBD, related to an increased risk of cardiovascular diseases. All the significant association observed in this manuscript were of small magnitude, indicating than other factors in addition to the one hereby studied contribute to explain the variance in SRBD.

## 1. Introduction

The International Classification of Sleep Disorders [1] divides sleep-related breathing disorders (SRBD) into a wide range of breathing abnormalities such as obstructive sleep apnea (OSA), central sleep apnea (CSA) syndromes, sleep-related hypoventilation and sleep-related hypoxemia disorders [2]. In pediatrics, the term SRBD also includes a variety of pathologies ranging from upper airway resistance syndrome and primary snoring to OSA-hypopnea syndrome [3]. SRBD are considered a major public health concern due to the high prevalence, closely related to obesity [4]. In childhood, SRBD are relatively common, with a prevalence of 12% [5]. More specifically, in a general population of children with 6 to 8 years, 10% were affected by SRBD [6]. Further, the prevalence of OSA is about 1% to 6% whilst the prevalence of habitual snoring range from 1% to 27%, in pediatric population [7,8,9,10].

It has been shown that SRBD often coexist with some morbidities related to the cardiovascular system (e.g., elevated blood pressure, and pulmonary hypertension) and the central nervous system (e.g., hyperactivity, excessive daytime sleepiness, cognitive deficits/academic problems, and behavioural problems), in childhood [11]. Specifically, current literature in children have shown that SRBD were associated with cardiovascular comorbidities and they impacted on sympathetic activity and, left and right, heart function [12]. Furthermore, OSA was related to an increased risk of resistant hypertension [13] and metabolic syndrome [14]. On the other hand, SRBD have been associated with multiple comorbidities such as poorer academic performance [15], lower certain aspects of behavioral functioning [16] and decreased parental-reported quality of life [11] at childhood. Therefore, studies examining the relationship between SRBD and health outcomes in children are needed.

Nowadays, obesity is one of the main public health-related problems, being considered one of the main risk factors for developing cardiovascular diseases, insulin resistance, hypertension and some types of cancer [17]. Pediatric obesity is associated with future mortality and morbidity, as well as with greater risk of cardiovascular diseases [18,19]. One study highlighted the importance of treating early overweight/obesity and SRBD in children to reduce the risk of future cardiovascular diseases [20]. Further, childhood obesity is another key risk factor for developing SRBD, especially in older children and adolescents. In fact, SRBD are presented in 26% to 33% of children with obesity [21] showing that the heavier you are, the higher the likelihood to suffer any SRBD [22,23,24,25,26,27]. Indeed, the prevalence of OSA, one of the most common causes of SRBD in children [7], ranges from 15% to 37% in non-obese children and from 33% to 76% in children with obesity depending on the definition of OSA, the age of the study sample, and the degree of obesity [28]. In a specific study of 12-year-old children with overweight/obesity, OSA prevalence was 44.6% [29]. In line with these assumptions, Mathew and Narang [30] concluded that children with overweight/obesity and adolescents could suffer OSA more frequently and may be more severe compared to lean children [30]. However, there is a need to deeply investigate how these factors are associated with risk of SRBD in childhood and, further, to study which of them, if any, is the best indicator of SRBD to ease its diagnostic.

Sedentary behaviour has been linked to SRBD [31]. For instance, many sedentary behaviours tend to be associated with an elevated risk of insomnia and sleep disturbance in the existing literature [32]. Indeed, low physical activity (PA) levels [33] and exercise capacity [34] have been associated to OSA. Likewise, most of the associated causes and comorbidities of SRBD are known to negatively impact cardiorespiratory fitness (CRF) [35].

Therefore, the aims of the present study were (i) to examine the association of sedentary behaviour, PA, CRF and body composition parameters with risk of SRBD; and (ii) to analyse which of these variables is the best explainer of SRBD in children with overweight/obesity.

## 2. Methods

### 2.1. Study Design and Participants

The present cross-sectional study is under the framework of the ActiveBrains Project. A detailed description of the study protocol, aims and methods has been published elsewhere [36]. A total of 110 children with overweight/obesity from Granada (south of Spain) were recruited. Recruitment was done at the Unit of Pediatrics of the ‘San Cecilio’ and ‘Virgen de las Nieves’ University Hospitals and health care centers of Granada (Spain). Additionally, the head teacher of both private and public schools of Granada were contacted and advertisements in the local media were published [36]. Baseline data collection was performed from November 2014 to February 2016. Of them, one child was excluded for difficulties shown in reading comprehension, and thus, a total 109 children (10.0 ± 1.1 years old, 45 girls) with overweight (*n* = 27) and obesity (*n* = 82) were included in the analyses. 

Parents or legal guardians were informed about the purpose of the study and parental written informed consents for all participants were obtained. This study was conducted according to the Declaration of Helsinki. The study protocol was approved by the Ethics Committee on Human Research (CEIH) of the University of Granada and was registered in ClinicalTrials.gov (identifier: NCT02295072).

### 2.2. Procedures and Measurements

#### 2.2.1. Sedentary Behaviour and Physical Activity

Television (TV) viewing time is known to be the most prevalent and common measure of leisure time sedentary behaviour [37,38]. TV viewing time was self-reported using the Spanish adaptation of the “Youth Activity Profile” questionnaire (YAP-S). The YAP-S was designed to be a self-administered 7-day recall questionnaire suitable for use in children to capture PA and sedentary behaviour [39]. The YAP questionnaire has been previously calibrated against accelerometer-determined sedentary behaviour and PA [39] and cross-validated in a different cohorts against accelerometer estimates as well [40,41].

The complete time engaged in sedentary time was objectively measured using wrist-worn accelerometers (ActiGraph GT3X+, Pensacola, FL, USA). Briefly, participants were requested to wear the accelerometers in their non-dominant wrist for the complete 24 h of the day for 7 consecutive days. Accelerometers were initialized at a sampling frequency of 100 Hz. Then, the raw accelerations were processed in the GGIR package v. 1.5–21 (https://cran.r-project.org/web/packages/GGIR/) in the R software (http://cran.r-project.org). Standard procedures were used to detect and impute non-wear time [42]. Sleep period time was detected using an automatized algorithm guided by reported sleep onset and waking-up times by the participants [43]. Euclidean norm minus one G (ENMO, 1 G~9.8 m/s^2^) was calculated as (i.e., $\sqrt{x^{2}+y^{2}+z^{2}}$ − 1 *G*) over 5 s epochs after auto-calibration of the raw acceleration [44,45]. Finally, the cut points of Hildebrand et al. [46] were used to classify ENMO into sedentary time and moderate-to-vigorous physical activity (MVPA). Daily means were used for the analyses (min/day). 

#### 2.2.2. Cardiorespiratory Fitness

CRF was assessed with the 20-m shuttle-run test as suggested in the ALPHA (Assessing Levels of Physical fitness and Health in Adolescents) health-related fitness test battery for youth [47]. It is known that this fitness test battery is feasible, reliable, valid and related to health in children [48,49,50]. Briefly, the test consists in running back and forth, between two lines 20 m apart, following an audio signal. Participants finished the test when they did not reach the line in two consecutive trials or stopped due to fatigue. We recorded the last completed lap and then, we translated it into an estimated maximal oxygen consumption measure (VO_2_max, mL/kg/min) with the formula described by Léger [51].

#### 2.2.3. Body Composition Parameters

Weight was assessed with an electronic scale (SECA 861, Hamburg, Germany), while height was measured using a precision stadiometer (SECA 225, Hamburg, Germany) to the nearest 0.1 kg and cm, respectively. Body mass index (BMI) was calculated as weight/height squared (kg/m^2^). Children were classified as overweight or obese using the sex- and age-specific international BMI cut offs proposed by the World Obesity Federation [52,53]. Waist circumference (WC) was measured using a homologated flexible steel tape (SECA, Hamburg, Germany). Weight, height, and WC were measured twice, and the average was considered for the analyses. Fat mass (kg) and lean mass (kg) were assessed by Dual-energy X-ray absorptiometry (DXA, Discovery densitometer from Hologic), following protocols used in previous studies [54,55]. Participants’ fat mass index (FMI) and lean mass index (LMI) were calculated by dividing their outcome (i.e., fat mass or lean mass) by their squared height (kg/m^2^).

#### 2.2.4. Sleep-Related Breathing Disorders

SRBD were evaluated with the Spanish version of the Pediatric Sleep Questionnaire (PSQ), which has shown high reliability and internal consistency [56]. This questionnaire was completed by parents, who should rate each item according to their child’s usual sleep habits. This questionnaire has been validated for the identification of SRBD risk and covers a wide spectrum of breathing abnormalities ranging from primary snoring to central sleep apnea, sleep-related hypoventilation, or OSA [1].

A SRBD scale was calculated from the 22 items of the questionnaire. The SRBD scale consists of 22 closed response question-items of the reduced version of the PSQ, which are divided into three domains as follows [57]: snoring (nine items), sleepiness (seven items) and behaviour (six items). The answer options for each item include “yes”, “no”, and “I do not know”. In the last domain of the questionnaire, the answers were structured in “never”, “sometimes”, “often” or “almost always”. In order to be consistent with these answer options throughout the questionnaire, one of the sections was re-categorized as follows: “never” and “sometimes” answers were categorized as “no”, whereas “often” and “almost always” answers were categorized as “yes”. The overall scale was calculated as the sum of the affirmative answers divided by 22. Occasional missing answers or responses of “I do not know” were discounted from the denominator when calculating these proportions. Scores > 0.33 were considered suggestive of high risk for a pediatric SRBD [57].

#### 2.2.5. Covariates

Age, sex, and maternal education level were included as covariates. Maternal education was obtained by a self-reported questionnaire about the maximum education level achieved. Mothers’ responses were classified as primary school, secondary school, and university degree completed.

### 2.3. Statistical Analysis

Descriptive characteristics are presented as means and standard deviations for continuous variables and as frequency and percentages for categorical variables. All variables were checked for normality. Sex differences were examined by *t*-tests and chi-squared tests for continuous and categorical variables, respectively.

Pearson’s bivariate correlations were used to examine the association of sedentary behaviour, PA, CRF and body composition variables with SRBD scale. Hierarchical stepwise regressions were used to indicate the main explanatory variables of SRBD among sedentary behaviour, PA, CRF and body composition parameters. All the variables (i.e., age, sex, maternal education, TV viewing time, sedentary time, MVPA, CRF, BMI, FMI, LMI and WC) were included together in the regression. We calculated variance inflation factors (VIF) for the models performed and only considered those with VIF < 10 to account for the risk of collinearity among explanatory variables.

Additionally, participants were classified into low (≤1 h/day) and high (>1 h/day) TV viewing groups. Although 2-h TV viewing has been related to negative health consequences [38], we considered that our sample, composed exclusively of children with overweight/obesity, could experience health consequences even with just 1 h of TV viewing per day. Differences between groups were analyzed with a one-way analysis of covariance (ANCOVA) adjusted for age, sex, and maternal education.

All analyses were performed using the Statistical Package for Social Science (IBM SPSS Statistics for Windows, version 22.0, Armonk, NY, USA). The level of significance was set at *p* < 0.05.

## 3. Results

Descriptive characteristics of the participants stratified by sex are presented in Table 1.

Pearson’s bivariate correlation analyses are presented in Table 2. In regard to sedentary behaviours, TV viewing time was positively associated with SRBD (*r* = 0.222, *p* = 0.021) whilst sedentary time was not (*r* = 0.129, *p* = 0.193). MVPA was not significantly associated with SRBD (*r* = 0.054, *p* = 0.585). CRF was negatively correlated with SRBD (*r* = −0.210, *p* = 0.030). Finally, all body composition parameters were positively associated with SRBD (r ranging from 0.191 to 0.223, all *p* ≤ 0.047), except FMI (*r* = 0.153, *p* = 0.114).

Hierarchical stepwise regression for the association of sedentary behaviour, PA, CRF and body composition parameters with SRBD is presented in Table 3. BMI was the predictor which explained the largest proportion of the variance in SRBD (*r*^2^ = 0.063, *p* = 0.011). Among the rest of explainers, TV viewing time was the only one added to this model with a significant contribution (*r*^2^ change = 0.048; *p* = 0.022).

It is important to note that all the significant association observed in this manuscript were of small magnitude, indicating than other factors in addition to the one hereby studied contribute to explain the variance in SRBD.

**Figure 1:**
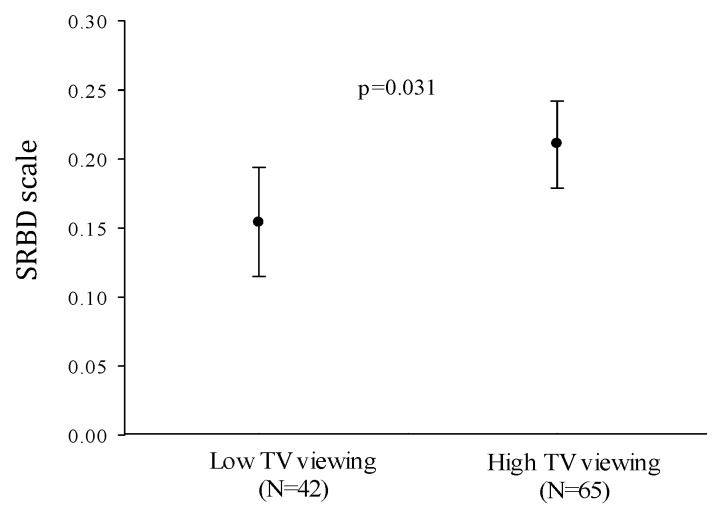
Differences in sleep-related breathing disorders between low and high TV viewing categories. Error bars represent mean and 95% confidence interval. Analyses are adjusted for age, sex, and maternal education. Low TV viewing: ≤ 1 h/day; High TV viewing: > 1 h/day. TV: television. SRBD: sleep-related breathing disorders.

ANCOVA analyses showed that children viewing TV for more than 1 h/day presented higher SRBD values than their peers after adjustment for age, sex and maternal education (mean difference = 0.057; *p* = 0.031; *F* = 4.779; df = 1) (Figure 1).

## 4. Discussion

The main findings of this study were: (1) TV viewing time and BMI parameters were positively associated with risk of SRBD. CRF was negatively associated with risk of SRBD; and (2) among the variables studied, BMI and TV viewing time were the best explainers of SRBD in children with overweight/obesity, explaining together around 11% of the variance in SRBD.

A previous study has shown that sedentary behaviour such as TV viewing (TV mobile screen watching) is strongly related to childhood obesity [58]. Additionally, it is shown that higher durations of TV viewing and screen time were associated with poorer body composition [59]. Our findings contribute to the existing knowledge by suggesting that those children with overweight/obesity who spend more than 1 h viewing TV also showed higher SRBD values. Likewise, TV viewing was a stronger indicator of SRBD than objectively assessed sedentary time and MVPA. Paavonen et al. [60] found that TV viewing was related to shorter sleep duration, sleeping disorders, and overall sleep disturbances in 5 to 6-year-old normal weight children. Our findings complement these ones by suggesting the same association in older children, i.e., 8.5 to 12-year-olds. Of note, a cut point of 2 h of TV viewing time is usually considered as being malign for health [38]. However, since our participants were children with overweight/obesity, we assumed that lower doses of TV viewing time are especially harmful for this sample, and so, 1 h of TV viewing time was considered to compare subgroups in our study. Concerning sedentary time measured through accelerometers, we did not observe any association with SRBD. The lack of capacity to discern between sedentary activities by the accelerometer could partially explain this finding. Similarly, no relationship between MVPA and SRBD was found. Although we have not found studies testing the associations between objectively-assessed MVPA and SRBD, it seems that 40 min per day of vigorous aerobic exercise could improve symptoms of SRBD in overweight 7 to 11-year-old children [61].

CRF is an important health marker, not only in adults but also in children and adolescents [62]. In consonance with previous studies [35,63], we found that CRF was negatively associated with SRBD values. Although this association was weak, our sample size was limited; thus, further studies with larger sample sizes are needed to estimate the real effect size of this association. For instance, Stojek et al. [63] found that higher levels of CRF were closely linked to less SRBD in inactive children with overweight/obesity aged 7 to 11 years. A possible explanation could be that the high oxygen demanding produced by CRF could improve the efficiency of the children’s cardiorespiratory system and, therefore, reduce SRBD.

Current literature provides evidence that children with overweight/obesity commonly present SRBD [22,23,24,25,26,27]. Longitudinal studies showed that overweight/obesity status in adolescents was strongly associated with increased mortality in adulthood due to cardiovascular diseases or other obesity-related risk factors [64]. One study found that a higher body fat percentage at the age of 6 to 8 years was associated with a higher risk of having SRBD 2 years later [65]. Verhulst et al. [24] found that fat distribution, as represented by WC, waist-to-hip ratio, or fat mass percentage were associated with central sleep apnea but not with OSA in children with overweight/obesity. In concordance with our findings, a questionnaire study in children who were on average 9 years old, observed that WC was correlated with risk of SRBD [66]. Additionally, WC was associated with all levels of SRBD [25] and an increased amount of visceral fat was associated with serious manifestations of SRBD [67] in a population sample of children. Likewise, Bhatia et al. [68] observed that BMI was positively associated with sleep problems (i.e., total arousal index and desaturation index) in children with overweight/obesity. In regard to DXA measures, they found that total body fat mass and trunk fat mass were related to some SRBD indicators, but they did not included FMI among their measures. Specifically, they concluded that the value of fat mass to study SRBD depends in the distribution of the fat over the body, which could explain the lack of association that we found after considering height when calculate FMI. In contrast, our results suggested that LMI could be a better indicator of SRBD than FMI. A possible explanation could be that children with more LMI have higher obesity levels since they have to carry more fat and also have bigger nocturnal work of breathing and, thus, they develop more lean mass. Furthermore, sarcopenic obesity has been associated with SRBD in adults, but not obesity in general. The hypothesis is that an excess of fat is a predictor of SRBD only when the lean mass is low [69,70,71]. However, in children, loss of lean mass is not a common problem, and little has been studied about body composition in relation to SRBD. Further studies using gold-standard methods for body composition parameters are needed to contrast our findings in children with overweight/obesity.

BMI has been very criticized for its lack of ability to distinguish between fat and lean tissues, but also a recent study showed that this index is better in the prediction of mortality than fat mass measured by a gold-standard method [72]. In regard to SRBD, we found something similar; BMI was a stronger explainer of SRBD than any other body composition outcome measured by a gold-standard (i.e., DXA). Other body composition parameters such as LMI or WC were associated with SRBD, but they were unable to explain a proportion of variance in SRBD more than those already explained by BMI. Andersen et al. found that an increased BMI was associated with increased apnea-hypopnea index and significantly risk of having OSA in children aged 7 to 18 years with overweight/obesity [29]. These results differ from those provided by Carotenuto et al. [66], who suggested that visceral fat (expressed as WC) was a more reliable measure than total adiposity (expressed as BMI) to predict the risk for developing SRBD in children and adolescents with obesity. Differences in study samples (i.e., participants’ age) and methodology (i.e., different questionnaires) might be responsible for the differences between these findings and ours. In addition, they only included children with obesity, while we also considered overweight children, which could partially explain the signification of BMI in our sample compared to the sample of Carotenuto et al. [66]. More studies are needed for a better understanding of the relationships between body composition and SRBD in children with overweight/obesity.

Among the explanatory variables of SRBD included in the present study (i.e., sedentary behaviour, PA, CRF and body composition parameters), we found that BMI and TV viewing time were the strongest ones, explaining the largest proportion of the variance in SRBD. None of the rest of outcomes contributed to the explanation already shown by BMI and TV viewing time. Although more studies are needed to confirm this, these findings suggest that interventions oriented to reduce BMI and TV viewing time through the modification of lifestyle habits and practices of physical exercise could be useful for the treatment of SRBD in children with overweight/obesity, even if the association is not strong enough.

The high prevalence of obesity and its health-related problems, such as SRBD, in the current pediatric population makes crucial the investigation on environmental factors associated with this disorder. Previous research has shown links of sedentary behaviour, PA and body composition parameters with SRBD in pediatric population. Our study complements the current knowledge by: (1) confirming these findings, despite the weak link, and adding more information in the literature due to the lack of knowledge on this topic, and (2) testing which of the factors could be of predominant interest to treat SRBD in children with overweight/obesity. However, only 11% of the variation in SRBD was explained by the predictors included. There is an important amount of the variation which is explained by factors not included in our models. Whether this 11% is enough to help in the diagnostic of SRBD from early ages is something to be investigated in future research.

Some limitations of the present study need to be acknowledged. Firstly, this is a cross-sectional study; hence, it does not allow us to infer causality for any of the associations studied. Secondly, a gold standard was not used to identify risk of SRBD and our data were obtained from parental questionnaires instead of polysomnographic data; therefore, its accuracy in evaluating SRBD is limited. Thirdly, the no inclusion of normal weight children cannot allow us the comparison between different weight status populations. Fourthly, our sample size was limited; thus, even more powerful studies with larger sample sizes are needed to confirm or contrast our findings. Caution is advised with the interpretation of these findings regarding younger ages, when other factors may be major determinants of SRBD (e.g., adenotonsillar). Finally, there could be other potential explanatory factors for SRBD in addition to those hereby studied that need to be investigated in future studies. The strengths of this study were: (1) the inclusion of objectively measured, sedentary time, and PA with accelerometry; (2) the inclusion of reliable, valid, and related to health field-based fitness tests in children [48,49,50] and (3) the reliable and valid measures of body composition parameters.

## 5. Conclusions

Our results suggest that sedentary time, CRF, and body composition parameters were associated with risk of SRBD in children with overweight/obesity—TV viewing time and BMI being the strongest explainers of SRBD. This study supports the notion that higher body weight status negatively influences SRBD and adds that unhealthy behaviours such as high TV viewing time could independently contribute to worsen SRBD. PA interventions focused on reducing sedentary behaviours such as TV viewing time and improving both CRF and body composition parameters could be a good strategy to treat SRBD prevalence in children with overweight/obesity and to reduce cardiovascular diseases risk in adulthood. All the significant association observed in this manuscript were of small magnitude, indicating that other factors in addition to the one hereby studied contribute to explain the variance in SRBD. Further randomized controlled trials studies are needed in order to contrast or corroborate our findings.

## Figures and Tables

**Table 1:** Descriptive characteristics of the participants.

	All	Boys	Girls
*n* (%)	109 (100)	64 (59)	45 (41)
Age (years)	10.0 ± 1.1	10.2 ± 1.1	9.9 ± 1.1
Sedentary behaviour			
Television viewing time, *n* (%) (*n* = 107)			
None	1 (1)	0 (0)	1 (2)
<1 h/day	41 (38)	24 (38)	17 (39)
1–2 h/day	42 (39)	22 (34)	20 (47)
<2–3 h/day	13 (12)	8 (12)	5 (12)
>3 h/day	10 (9)	10 (16)	0 (0)
Sedentary time (min/day) (*n* = 103)	520.1 ± 54.7	521.3 ± 52.0	518.4 ± 58.9
Physical activity			
Moderate-to-vigorous physical activity (min/day) (*n* = 103)	51.4 ± 20.1	59.1 ± 21.0	40.2 ± 11.7
Cardiorespiratory fitness			
20 m shuttle run test (laps)	16.0 ± 7.7	17.2 ± 8.1	14.4 ± 6.9
20 m shuttle run test (VO_2max_, mL/kg/min) ^a^	40.7 ± 2.7	40.8 ± 2.7	40.6 ± 2.7
Body composition parameters			
Weight (kg)	56.2 ± 11.2	57.1 ± 11.2	54.9 ± 11.3
Height (cm)	144.2 ± 8.4	145.0 ± 8.0	143.1 ± 9.0
Body mass index (kg/m^2^)	26.8 ± 3.6	27.0 ± 3.7	26.6 ± 3.5
Fat mass index (kg/m^2^)	11.8 ± 2.9	11.5 ± 2.9	12.1 ± 2.8
Lean mass index (kg/m^2^)	14.0 ± 1.4	14.3 ±1.3	13.5 ± 1.4
Waist circumference (cm)	90.2 ± 9.9	91.3 ± 9.4	88.7 ± 10.5
Sleep-related breathing disorders			
SRBD scale (0 to 1)	0.2 ± 0.1	0.2 ± 0.1	0.2 ± 0.1
Presence of SRBD, *n* (%)	17(16)	10 (16)	7 (16)

Data are presented as mean ± standard deviation or frequency (%). SRBD: Sleep-related breathing disorders, VO_2max_: maximum oxygen consumption. ^a^ 20 m shuttle run test (VO_2max_) was estimated from the 20-m shuttle run test by the formula described by Léger et al. [51].

**Table 2:** Pearson’s bivariate correlation coefficients of sedentary behaviour, physical activity, cardiorespiratory fitness, and body composition parameters with sleep-related breathing disorders scale.

	SRBD Scale (0 to 1)	
	*r*	*p*
Sedentary behaviour		
Television viewing time (h/day)	0.222	**0.021**
Sedentary time (min/day)	0.129	0.193
Physical activity		
Moderate-to-vigorous physical activity (min/day)	0.054	0.585
Cardiorespiratory fitness (VO_2max_ mL/kg/min) ^a^	−0.210	**0.030**
Body composition parameters		
Body mass index (kg/m^2^)	0.209	**0.029**
Fat mass index (kg/m^2^)	0.153	0.114
Lean mass index (kg/m^2^)	0.223	**0.020**
Waist circumference (cm)	0.191	**0.047**

Statistically significant values (*p* < 0.05) are shown in bold. SRBD: sleep-related breathing disorders. ^a^ Cardiorespiratory fitness (VO_2max_) was estimated from the 20-m shuttle run test by the formula described by Léger et al. [51].

**Table 3:** Hierarchical stepwise regression for the association of sedentary behaviour, physical activity, cardiorespiratory fitness, and body composition parameters with sleep-related breathing disorders scale (*n* = 102).

	β	*p*-Value	Change *R*^2^	*R* ^2^
Model 1			0.063	0.063
Body mass index	0.251	**0.011**		
Model 2			0.048	0.111
Body mass index	0.249	**0.010**		
Television viewing time	0.220	**0.022**		

β = standardized coefficients. Statistically significant values (*p* < 0.05) are shown in bold. Variables entered in the stepwise models: age, sex, maternal education, television viewing time, sedentary time, moderate-to-vigorous physical activity, cardiorespiratory fitness, body mass index, fat mass index, lean mass index, and waist circumference.
